# Genistein inhibits proliferation of colon cancer cells by attenuating a negative effect of epidermal growth factor on tumor suppressor FOXO3 activity

**DOI:** 10.1186/1471-2407-11-219

**Published:** 2011-06-03

**Authors:** Wentao Qi, Christopher R Weber, Kaarin Wasland, Suzana D Savkovic

**Affiliations:** 1 Department of Medicine, Division of Gastroenterology; NorthShore University Research Institute, Evanston, IL 60201, USA; 2 Department of Pathology, The University of Chicago; Chicago, IL 60637, USA

**Keywords:** Genistein, EGF, FOXO3, proliferation, colon cancer

## Abstract

**Background:**

Soy consumption is associated with a lower incidence of colon cancer which is believed to be mediated by one of its of components, genistein. Genistein may inhibit cancer progression by inducing apoptosis or inhibiting proliferation, but mechanisms are not well understood. Epidermal growth factor (EGF)-induced proliferation of colon cancer cells plays an important role in colon cancer progression and is mediated by loss of tumor suppressor FOXO3 activity. The aim of this study was to assess if genistein exerts anti-proliferative properties by attenuating the negative effect of EGF on FOXO3 activity.

**Methods:**

The effect of genistein on proliferation stimulated by EGF-mediated loss of FOXO3 was examined in human colonic cancer HT-29 cells. EGF-induced FOXO3 phosphorylation and translocation were assessed in the presence of genistein. EGF-mediated loss of FOXO3 interactions with p53 (co-immunoprecipitation) and promoter of p27kip1 (ChIP assay) were examined in presence of genistein in cells with mutated p53 (HT-29) and wild type p53 (HCT116). Silencing of p53 determined activity of FOXO3 when it is bound to p53.

**Results:**

Genistein inhibited EGF-induced proliferation, while favoring dephosphorylation and nuclear retention of FOXO3 (active state) in colon cancer cells. Upstream of FOXO3, genistein acts via the PI3K/Akt pathway to inhibit EGF-stimulated FOXO3 phosphorylation (i.e. favors active state). Downstream, EGF-induced disassociation of FOXO3 from mutated tumor suppressor p53, but not wild type p53, is inhibited by genistein favoring FOXO3-p53(mut) interactions with the promoter of the cell cycle inhibitor p27kip1 in colon cancer cells. Thus, the FOXO3-p53(mut) complex leads to elevated p27kip1 expression and promotes cell cycle arrest.

**Conclusion:**

These novel anti-proliferative mechanisms of genistein suggest a possible role of combining genistein with other chemoreceptive agents for the treatment of colon cancer.

## Background

Soy consumption is associated with a lower incidence of cancer in Asian countries [1,2]. Although these epidemiological studies are correlative, it has been hypothesized that soy compounds may have anti-cancer properties. Indeed numerous studies have shown a prominent component of soy, genistein, has anticancer properties [3-5], and the mechanism whereby genistein exerts anticancer effects has been the subject of considerable interest.

It has been shown that a synthetic analogue of the genistein, phenoxodiol, significantly reduced colonic tumor growth through inhibitory effects on the immune system [6]. Genistein effectively suppresses the growth of colon cancer cells [7] by attenuating activity of the PI3K/Akt pathway [7-9], which is known to be critical in the regulation of colon cancer progression [10,11]. Additionally, genistein affects the Wnt signaling pathway in colon cancer cells, which is known to be important to colon tumorigenesis [12] by inducing Wnt5a expression [13]. Finally, a recent study demonstrated that in colon cancer cells genistein affect the expression of estrogen receptor and some tumor suppressor genes [14,15] supporting a role of membrane receptors and tumor suppressors in antiproliferative effects of genistein.

In human colon cancer EGF receptor (EGFR) expression and activity are increased [16,17], and targeting this receptor has played an increasing therapeutic role [18]. We have demonstrated that proliferation of colon cancer cells, stimulated with signals from EGFR, is mediated by loss of tumor suppressor FOXO3 activity [19]. EGF attenuates FOXO3 activity via the PI3K/Akt pathway and results in loss of cell cycle arrest and enhanced proliferation [19]. When activate (dephopshorylated), FOXO3 is localized in the nucleus and binds to DNA or other transcriptional factors regulating the expression of specific target genes involved in control of cell cycle progression, the mitotic program, or induction of apoptosis [20]. The effect of genistein on EGF-mediated loss of FOXO3 activity and associated colon cell proliferation has not been determined. We hypothesize that anti-proliferative properties of genistein in colon cancer cells are mediated by inhibition of the negative effect of EGF on FOXO3 activity, thus promoting cell cycle arrest.

This study demonstrates a new anti-proliferative mechanism of genistein mediated by inhibiting the negative effect of EGF on tumor suppressor FOXO3, which favors the interaction of FOXO3 with mutated p53 in colon cancer cells. The FOXO3-p53(mut) complex binds to the promoter of p27kip1, causing increased p27kip1 expression and subsequent induction of cell cycle arrest in colon cancer cells. This is a novel anti-proliferative mechanism and is relevant to designing novel therapeutic agents, analogous to genistein, which may be used to treat colon cancer.

## Methods

### Cell Culture

HT-29 colon cancer cells (American Type Culture Collection (ATCC), Manassas, VA), carrying mutation in tumor suppressor p53, and HCT116, with wild type p53, were grown in McCoy's 5A medium (Sigma-Aldrich, Saint Louis, MO) containing 10% FBS (Gibco) at 37°C and 5% CO_2_. Monolayers were kept in McCoy's 5A media without serum for 20-24 h before experiments.

### Treatment

To examine the effects of genistein on proliferation, cells were incubated with 10-150 μM genistein (LC Laboratories, Woburn, MA) for 48 hours. To examine the effects of genistein on induced FOXO3 phosphorylation, translocation, interaction with p53, and binding to p27kip1 promoter, monolayers were treated with EGF (100 ng/ml) (Sigma-Aldrich) with and without mild concentration of genistein (50 μM) for 48 hours [21,22]. During EGF and genistein treatment, cells were placed in serum-free and antibiotic-free medium.

### Immunofluorescent Staining

To determine the inhibitory effect of genistein on EGF-induced FOXO3 translocation from the nucleus to the cytosol immunofluorescent staining was performed. Monolayers were fixed with 3.7% paraformaldehyde and permeabilized with 0.2% Triton X-100. For staining, anti-FOXO3 primary antibody (Cell Signaling, Danvers, MA) and Alexa 488 conjugated secondary antibody were used (Molecular Probes-Invitrogen, Carlsbad, CA), as previously described [19,23,24]. After washing with PBS, coverslips were mounted using Prolong Gold antifade reagent (Molecular Probes), and images were captured with a Nikon Confocal Microscope C1 and analyzed with EZ-C1 software (Nikon, Tokyo, Japan).

### Protein Extraction

Total protein was extracted using a lysis buffer (Cell Signaling, Danvers, MA) with a protease inhibitor cocktail (Sigma-Aldrich), and protein concentration was determined by Bradford assay (Bio-Rad, Hercules, CA). The protein extracts were stored at -20°C until further processing.

### Immunoblot

Equal amounts of protein (40 μg) were separated by SDS-PAGE and transferred to nitrocellulose membranes by voltage gradient transfer (Bio-Rad). Prepared blots were blocked and detection was performed using specific antibodies for total FOXO3 (Cell Signaling Technology, Danvers, MA), phosphorylated FOXO3 at Thr 32 (Upstate Biotechnology), pAkt (Cell Signaling), p27kip1 (Cell Signaling), actin, EGFR, pEGFR, and p53 (Santa Cruz Biotechnology, Santa Cruz, CA). After washing, the blots were incubated with horseradish peroxidase linked secondary antibodies (Cell Signaling, Danvers, MA), and detection was achieved with ECL plus western blotting detection reagents (GE Healthcare, Buckinghamshire, United Kingdom). Intensity of the bands was quantified by optical densitometry using Labworks 4.6 Image Acquisition and Analysis Software (UVP, Cambridge, UK), and was calculated as percentage of changes relative to control.

### Co-Immunoprecipitation

The effect of genistein on FOXO3-p53 incitation was assessed by co-immunoprecipitation. One milligram of whole cell lysate was incubated with 10 μg of mouse anti-FOXO3 antibody (Cell Signaling) and protein A beads overnight at 4°C. Immunoprecipitates were washed five times with lysis buffer, separated by SDS-PAGE, and transferred to membranes. Immunoblot analysis was performed with anti-p53 antibody from rabbit (Santa Cruz Biotechnology) to prevent cross-reaction. IgG antibody from mouse was used as a negative control.

### Chromatin Immunoprecipitation (ChIP) Assay

The effect of genistein on FOXO3 binding to p27kip1 promoter was examined by ChIP assay according to the manufacturer's instructions (Millipore, Temecula, CA). After cross-linking with 1% formaldehyde, the cells were incubated in lysis buffer and sonicated to cut DNA (200 to 1000 bp). Aliquots (20 μl) from each sample were held separately for use as "input DNA" in PCR analysis. Equal amounts of protein were incubated with FOXO3 (Cell Signaling) or p53 (Santa Cruz) antibodies at 4°C overnight, and the complexes comprised of DNA-protein were pelleted with protein G-agarose. After reversing the immunoprecipitated complexes and input aliquots with 5 M NaCl at 65°C for 4 hours, protein was separated from DNA using proteinase K. Extracted DNA (phenol/chloroform) was amplified using primers from p27kip1 promoter (forward: 5'-GTC CCT TCC AGC TGT CAC AT-3'; reverse, 5'-GGA AAC CAA CCT TCC GTT CT-3'). Input represents PCR amplification of DNA from cell lysate before immunoprecipitation with the primers used to amplify the p27kip1 promoter and β-actin (forward, 5'-CCA CAC TGT GCC CAT CTA CG-3'; reverse, 5'- AGG ATC TTC ATG AGG TAG TCA GTC AG-3').

### Cell Proliferation Assays

An inhibitory effect of genistein on proliferation of colon cancer cell lines was detected using the MTS assay (Promega; Medison, WI). Cells grown in regular media were plated on 96-well plates (5000 cells per well) and after 48 hours of incubation with the experimental compounds, part of the medium was removed, and MTS solution was added for another 3 hours at 37°C. A water-soluble formazan product converts from MTS and was detected at 490 nm using a SPECTRAmax Plus Microplate Reader (Molecular Devices, Sunnyvale, CA). Results obtained at 490 nm were converted to percentile changes relative to control.

### siRNA

Silencing p53 (siRNA) was utilized to determine its effect on FOXO3 activity in HT-29 cells. Cells were transfected with p53 siRNA (Santa Cruz Biotechnology) (GCAUGAACCGGAGGCCCAU) or negative-control (Invitrogen) using Lipofectamine RNAiMAX (Invitrogen). After 5 hours, transfection media was replaced with regular media containing genistein and protein was extracted 48 hours later.

### Statistical Analysis

Data were compared by a one-way analysis of variance and a Student's *t *test. The results are expressed as means ± standard deviation. Differences were considered significant at p < 0.05.

## Results

### Genistein inhibits EGF-induced proliferation, FOXO3 phosphorylation, and translocation in colon cancer cells

**Figure 1:**
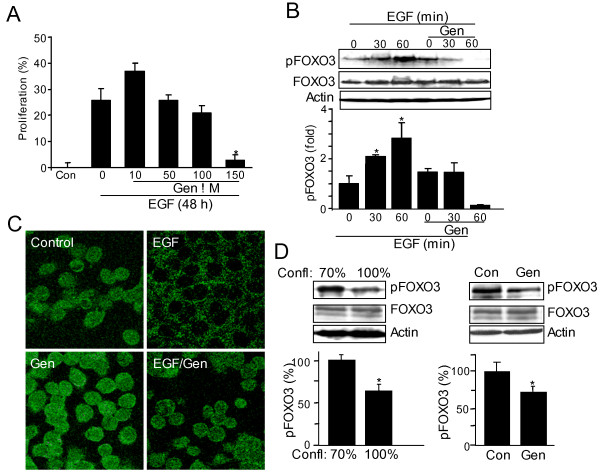
**Genistein inhibits EGF-induced proliferation and FOXO3 phosphorylation and translocation in colon cancer cells**. **(A) **HT-29 cells were stimulated with EGF in presence of genistein (10, 50, 100, and 150 μM) and assayed for proliferation 48 hours later by MTS. This experiment was repeated three independent times and graphed data are mean ± sd (n = 24, *p < 0.001). **(B) **Genistein inhibits EGF-induced FOXO3 phosphorylation. HT-29 cells treated with EGF, with and without genistein, were examined for phosphorylated FOXO3 at Thr32. Genistein inhibits EGF induced FOXO3 phosphorylation. Immunoblots were performed three independent times and graphs represent densitometric analysis means ± sd (n = 3, *p < 0.05). **(C) **Genistein inhibits EGF-induced translocation of FOXO3. Immunofluorescent staining of experimental monolayers revealed that EGF-induced FOXO3 translocation from the nucleus to the cytosol was inhibited by genistein. This experiment was repeated two times in triplicate. **(D) **Genistein inhibits FOXO3 phosphorylation in colon cancer cells. Equal amounts of protein extracted from 70% confluent (proliferative) and 100% confluent HT-29 monolayers were separated and immunoblotted with antibodies against phosphorylated FOXO3, total FOXO3, and actin. In proliferative HT-29 cells (70% confluent), the phosphorylated form of FOXO3 is increased relative to non-proliferative cells (100% confluent). HT-29 cells (70% confluent) were incubated with genistein for 24 hours and the status of phosphorylated FOXO3 was examined. Genistein attenuates phosphorylated FOXO3 in sub-confluent HT-29 cells. These experiments were repeated three times and graphs represent densitometric analysis (n = 8, *p < 0.05).

EGF promotes proliferation and is known to be critical to the progression of colon cancer [16-18]. Anti-proliferative properties of genistein, exerted by targeting different kinases of various proliferative pathways [9,25-28] were assessed on EGF-induced proliferation in colon cancer cells. EGF-induced proliferation of HT-29 cells was inhibited by genistein (Figure 1A), suggesting that genistein may affect the EGF pathway in colon cancer cells. We previously demonstrated that EGF-induced proliferation is mediated via loss of tumor suppressor FOXO3 activity [19]. In the presence of genistein, EGF-induced FOXO3 phosphorylation at Thr32 (inactivation) [19] was inhibited (Figure 1B), showing that genistein promotes FOXO3 activity. Active FOXO3 localizes to the nucleus and following phosphorylation by EGF, FOXO3 translocates to the cytosol [19]. Genistein inhibited EGF-induced FOXO3 translocation to the cytosol, and thus FOXO3 remained in the nucleus (Figure 1C). Moreover, the high basal level of phosphorylated FOXO3 (inactive) in sub-confluent HT-29 cells was significantly diminished by genistein (Figure 1D), further supporting that genistein promotes FOXO3 activity in proliferative colon cancer cells regardless of EGF stimulation. These data suggested that attenuation of EGF-induced proliferation by genistein is in part mediated by inhibition of FOXO3 phosphorylation (inactivation) and translocation to the cytosol in colon cancer cells (i.e. FOXO3 inactivation).

### Genistein inhibition of EGF-induced FOXO3 phosphorylation is mediated by the PI3K/Akt pathway

**Figure 2:**
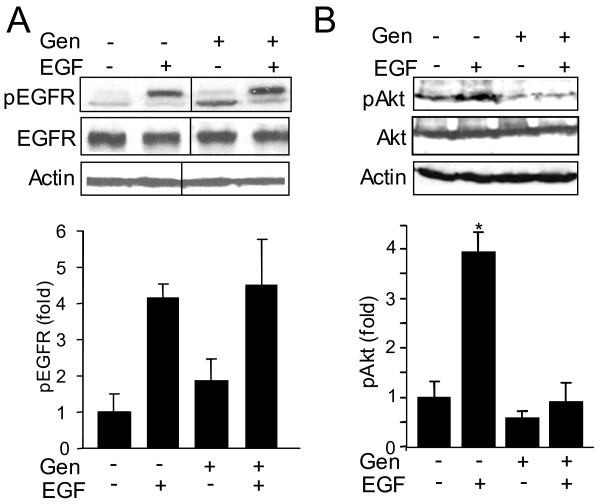
**Genistein inhibits FOXO3 phosphorylation via PI3K/Akt**. Total protein from HT-29 cells (control and EGF treated for 30 minutes) was examined for phosphorylated **(A) **EGFR and **(B) **Akt in the presence and absence of genistein. Genistein inhibits EGF induced Akt phosphorylation in HT-29 cells. The experiment was repeated in triplicate and graphs represent densitometric analysis (n = 3, *p < 0.05).

The above data show that genistein inhibits EGF-induced FOXO3 phosphorylation at Thr32, which is known to be a PI3K/Akt specific site [19]. Since PI3K/Akt is downstream of EGFR, we sought to examine whether genistein targets the EGFR or the PI3K/Akt pathway. Although genistein modestly increases basal pEGFR (at Ser1070), it did not affect expression and phosphorylation of the EGFR during EGF treatment (Figure 2A). Additionally, an EGF-induced 4-fold increase in Akt phosphorylation was diminished by genistein (Figure 2B). Also, genistein insignificantly decreased the basal level of pAkt. Thus we speculate genistein inhibits EGF-induced FOXO3 phosphorylation via the PI3K/Akt pathway.

### Genistein inhibits EGF-induced FOXO3 disassociation from the promoter of p27kip1 cell cycle inhibitor

**Figure 3:**
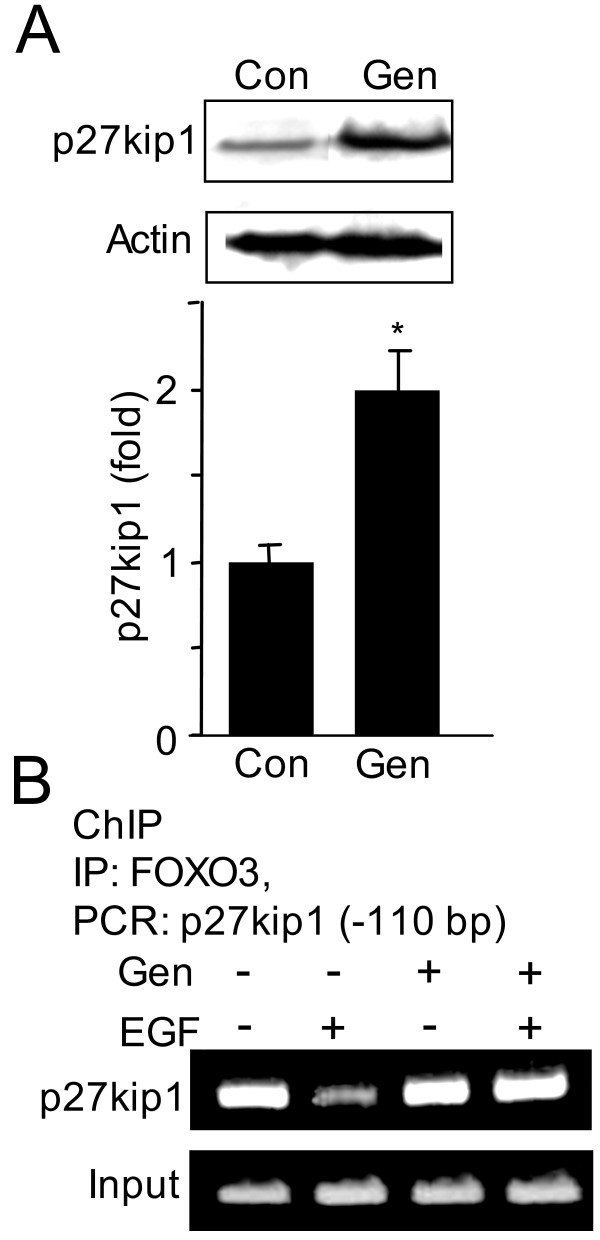
**Genistein increases p27kip1 expression and promotes FOXO3 binding to the p27kip1 promoter**. **(A) **Protein from control and genistein treated HT-29 cells was separated and immunoblotted for p27kip1. Immunoblots revealed that genistein increased p27kip1. The experiment was performed three independent times and quantified using densitometry (n = 3, *p < 0.05). **(B) **Genistein inhibits FOXO3 disassociation from p27kip1 promoter. ChIP assay was performed to determine FOXO3 binding to p27kip1 promoter (-110 bp). EGF-induced FOXO3 disassociation from p27kip1 promoter (-110 bp) was inhibited by genistein. Input represents PCR amplification of DNA from cell lysate prior to immunoprecipitation with the primers from p27kip1 promoter. This experiment was repeated two times.

Downstream, EGF treatment led to FOXO3 disassociation from the promoter for the cell cycle inhibitor p27kip1 [19]. Thus we assessed if genistein inhibits proliferation by preventing EGF-induced FOXO3 disassociation from p27kip1 promoter. In HT-29 cells genistein increased p27kip1 expression 2-fold (Figure 3A) and ChIP assay revealed that genistein inhibits EGF-induced FOXO3 disassociation from p27kip1 promoter (Figure 3B). Thus, we speculate that genistein promotes FOXO3 binding to the p27kip1 promoter, increasing p27kip1 expression, and ultimately leading to cell cycle arrest in colon cancer cells. Next, we sought to determine the mechanism whereby genistein promotes FOXO3 binding to the p27kip1 promoter.

### Genistein inhibits EGF-mediated disassociation of FOXO3 from p53(mut) tumor suppressor

**Figure 4:**
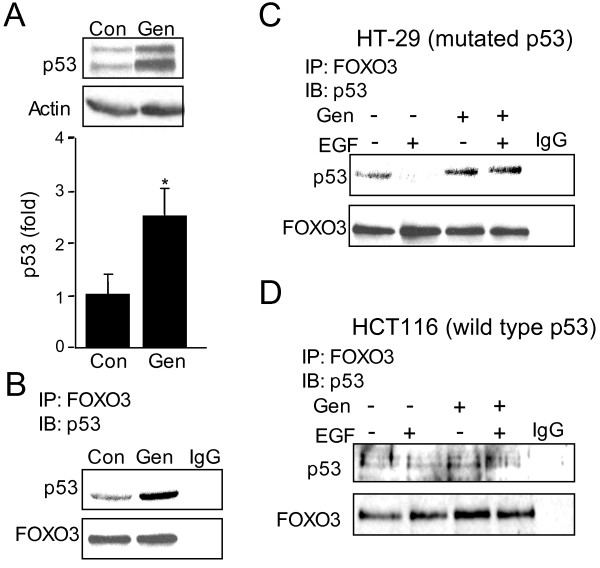
**Genistein increases FOXO3-p53(mut) interaction in HT-29 cells**. **(A) **Protein from control and genistein treated HT-29 cells was immunoblotted for p53. Genistein increased p53 expression 2.5-fold. The experiment was performed three independent times and quantified using densitometry (n = 3, *p < 0.05). **(B, C) **Protein from different experimental groups from HT-29 cells was immunoprecipitated with antibody against FOXO3 (mouse) and immunoblotted using antibody against p53 (rabbit). Co-immunoprecipitation revealed that genistein increased FOXO3-p53(mut) interaction and prevented EGF induced disassociation of p53 and FOXO3. IgG (mouse) was used as a negative control. Experiments were repeated three times. **(D) **Protein from HCT116 with wild type p53 was immunoprecipitated with anti-FOXO3 (mouse) and immunoblotted using antibody against p53 (rabbit). Nether EGF nor genistein affected FOXO3 interaction with wild type p53 in HCT116 cells.

Transcriptional activity of FOXO3 could be modulated though interactions with other transcriptional factors such as tumor suppressor p53 [29], which is known to be mutated in some colonic cancers and is critical to cancer progression [30-33]. We assessed if genistein affects interactions of FOXO3 with mutated p53, thus further affecting FOXO3 activity. Genistein increases expression of mutated p53 by 2.5-fold in HT-29 cells (Figure 4A), and co-immunoprecipitation demonstrated an increased interaction of p53(mut) with FOXO3 (Figure 4B). Moreover, co-immunoprecipitation shows that the FOXO3-p53(mut) complex is diminished during EGF treatment, while genistein reduces the effect of EGF (Figure 4C). Next, we assessed if FOXO3-p53 interactions are specific for mutated p53 using colonic HCT116 cells with wild type p53. In HCT116 cells, although FOXO3-p53 complex was found, EGF and genistein did not affect this interaction (Figure 4D), supporting that the interaction of FOXO3 with mutated p53 is targeted by genistein. Taken together these data show that the interaction between p53 and FOXO3, which is impaired by EGF, is promoted by genistein in HT-29 cells.

### Genistein mediated FOXO3 and p53(mut) interaction promotes FOXO3 activity on p27kip1 promoter

**Figure 5:**
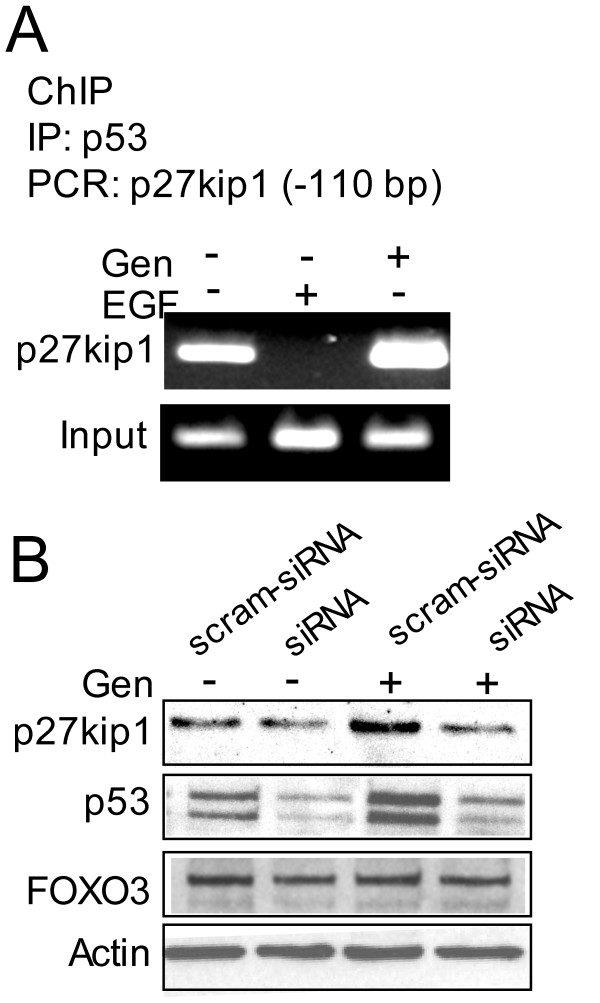
**The FOXO3-p53(mut) complex promotes p27kip1 expression**. **(A) **The protein-DNA complex was immunoprecipitated with antibody against p53 and DNA was amplified by conventional PCR with primers from the p27kip1 promoter region (ChIP assay) with input representing PCR amplification from lysate before immunoprecipitation with the same primers. The FOXO3-p53(mut) complex is bound to the p27kip1 promoter. **(B) **HT-29 cells transfected with p53 siRNA or scramble siRNA were treated with genistein and examined for p27kip1 by immunoblot. Silencing of p53 attenuates genistein-induced p27kip1 expression. These experiments were repeated two independent times.

**Figure 6:**
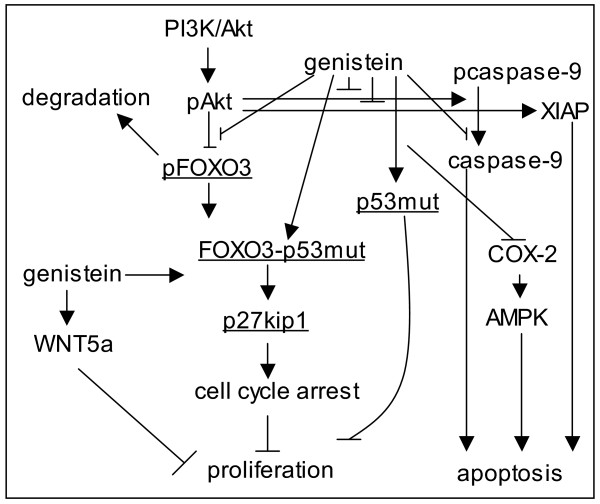
**Genistein inhibits proliferation in colon cancer cells by promoting FOXO3 activity**. Schematic representation of pathways targeted by genistein in colon cancer cells [7-9,13,21,50]. Genistein promotes FOXO3 activity by inhibiting PI3K/Akt and stimulating FOXO3 interaction with p53. Downstream, genistein-mediated FOXO3 activity increases p27kip1 expression, which promotes cell cycle arrest and leads to inhibition of proliferation.

The above data support that genistein increases FOXO3 binding to the p27kip1 promoter and also favors FOXO3 interactions with p53(mut). However, the p27kip1 promoter did not show putative p53 binding sites (GGACATGCCCGGGCATGTCC) [34,35] in the -200 bp regions where the FOXO3 binding site is located (-110 bp). Thus, we hypothesized that the FOXO3-p53(mut) complex could be found on the FOXO3 binding site of p27kip1 promoter. Using ChIP assay, performed by immunoprecitating protein-DNA complex with anti-p53 antibody, p53 was found to be present in the p27kip1 promoter within the FOXO3 binding region (Figure 5A). In HT-29 cells with silent p53, genistein did not increase p27kip1 expression (Figure 5B), supporting that p53(mut) positively regulates FOXO3 activity in the FOXO3-p53(mut) complex. The genistein-stimulated interaction between p53 and FOXO3 promotes FOXO3 activity and resistance to EGF, thus increasing expression of the p27kip1 cell cycle inhibitor (Figure 6).

## Discussion

Genistein, a predominant component of soy products, has been shown to have anti-cancer properties [2,3,5]. This study revealed a novel mechanism that genistein utilizes to inhibit proliferation. Proliferation of EGF treated colon cancer cells is mediated by loss of FOXO3 activity [19], and here we showed this pathway to be inhibited by genistein. Upstream, genistein inhibits EGF induced loss of FOXO3 activity by targeting the PI3K/Akt pathway. Downstream, genistein inhibits EGF-induced FOXO3 disassociation from p53(mut), which further promotes FOXO3 activity and leads to increased expression of the p27kip1 cell cycle inhibitor, which inhibits proliferation in colon cancer cells.

We demonstrated that one of the anti-proliferative mechanisms of genistein in colon cancer cells is to promote FOXO3 activity by inhibiting EGF-induced FOXO3 phosphorylation (inactivation) via the PI3K/Akt pathway. Active FOXO3 negatively regulates proliferation of colon cancer cells [36], and we showed that its inactivation is an essential step in EGF-mediated proliferation [19]. Although some studies demonstrated that high concentrations of genistein can downregulate EGFR in prostate cells [37], we showed that the concentration of genistein used for this study did not affect EGFR expression in colon cancer cells and had modest effects on activation of EGFR that are most likely non-specific. It has been shown that genistein inhibits EGF-stimulated serine, threonine, and tyrosine phosphorylation [38]. Also, genistein affects estrogen receptors [39], which are critical in colon cancer progression [14]. Therefore, we speculate, that genistein inhibit Akt independently of EGFR, by attenuating either kinase activity downstream of EGFR or blocking estrogen receptor. It has been previously demonstrated that genistein inhibits proliferation in colon cancer cells via PI3K/Akt [40], a pathway known to be critical to colon cancer progression [10,11,41], however downstream mechanisms were not well understood. This study demonstrated that genistein inhibits PI3K/Akt activation that leads to prevention of FOXO3 phosphorylation (inactivation) in colon cancer cells and revealed a new mechanism whereby genistein attenuates proliferation of colon cancer cells.

Active FOXO3 attenuates proliferation by upregulation of the cell cycle inhibitor p27kip1 [36,42], and we showed that EGF-induced FOXO3 disassociation from the p27kip1 promoter [19] is inhibited by genistein in colon cancer cells. In prostate and breast cancer cells, the anti-proliferative effects of genistein occur by increasing levels of the cell cycle inhibitor p27kip1 [43,44], but upstream mechanisms were not understood. Here we showed that genistein increases p27kip1 expression in colon cancer cells by promoting FOXO3 binding to the p27kip1 promoter. It is important to take into account that increased p27kip1 by genistein is most likely one of the mechanisms of inhibition of proliferation and that the other targeted molecules also play a role. Also, this study demonstrated that for increased p27kip1 expression, interaction between FOXO3 and mutated tumor suppressor p53 is required. In contrast to human lung cancer cells where genistein increased wild type but not mutated p53 [45], in colon cancer HT-29 cells we showed that genistein increased expression of mutated p53. Although, wild type p53 interacts with FOXO3 thereby decreasing its activity in the FOXO3-53 complex [29,46,47], this study demonstrated that mutated p53 increased FOXO3 activity in HT-29 cells. Additionally EGF treatment did not affect interactions between wild type p53 and FOXO3 further supporting that a mutation of p53 is most likely accountable for the genistein effect. Since a mutation of p53 is critical to colon cancer development [30-33], the anti-proliferative properties of genistein may relate to targeting mutated p53 and thus promoting FOXO3 activity and cell cycle arrest.

This study showed that genistein inhibits proliferation of colon cancer cells by attenuating a negative effect of EGF on tumor suppressor FOXO3 activity, thereby promoting FOXO3 interaction with mutated p53, which leads to expression of p27kip1 and cell cycle arrest. These findings support a potential role of genistein in combination with other chemopreventive agents [3,48,49] for the treatment of colon cancer.

## Conclusion

Genistein inhibits EGF-induced proliferation in colon cancer cells by promoting FOXO3 activity, targeting upstream the PI3K/Akt pathway, and stimulating downstream FOXO3 interaction with tumor suppressor p53mut. As a result of increased FOXO3 activity, expression of p27kip1 is elevated, which leads to cell cycle arrest. This is a new anti-proliferative mechanism for genistein and sets the foundation for the potential combined use of genistein with other chemoreceptive agents in the treatment of colon cancer.

## Competing interests

The authors declare that they have no competing interests.

## Authors' contributions

QW: Carried out and design the experiments, and participated in the preparation of figures. CW: Designed hypothesizes and participated in the preparation of the manuscript. KW: Performed initial studies finding this mechanism. SS: Envisioned the study, participated in its design, coordination and final manuscript preparation. All authors read and approved the final manuscript.

## Pre-publication history

The pre-publication history for this paper can be accessed here:

http://www.biomedcentral.com/1471-2407/11/219/prepub
